# Association between Subclinical Hypothyroidism and Glycemic Control in Older Adults in a Medical Center in Peru

**DOI:** 10.1155/2024/2156630

**Published:** 2024-09-03

**Authors:** Karen Quintanilla, Karla M. Joo, Hellen L. La Torre, Carlos D. Neyra-Rivera, Ericson L. Gutierrez, José F. Parodi, Fernando M. Runzer-Colmenares

**Affiliations:** ^1^ Universidad Científica del Sur Facultad de Ciencias de la Salud Carrera de Medicina Humana CHANGE Research Working Group, Lima, Peru; ^2^ Centro de Investigación del Envejecimiento (CIEN) Facultad de Medicina Humana Universidad San Martín de Porres, Lima, Peru; ^3^ Facultad de Ciencias de la Salud Universidad Tecnológica del Perú, Lima, Peru; ^4^ Universidad San Ignacio de Loyola Unidad de Investigación para la Generación y Síntesis de Evidencias en Salud, Lima, Peru

## Abstract

**Objective:**

To determine whether there is an association between subclinical hypothyroidism and glycemic control in older adults who received care at the “Centro Médico Naval” from 2010 to 2015.

**Methods:**

This retrospective analytical study analyzed a secondary database of the care of elderly in the study hospital. The sample was comprised of 1,385 older adults. To detect an association between variables, the *Poisson* regression with robust variance was used at a significance level of 95%. The analyses were carried out with the STATA 16 program.

**Results:**

Of the elderly 45.6% were between 71 and 80 years old; 58.4% were women and 43.8% had a normal body mass index. There was evidence of inadequate glycemic control in 8.1% and subclinical hypothyroidism in 15.2% of the elderly patients. Subclinical hypothyroidism was more frequent in the inadequate glycemic control vs. adequate glycemic control populations (41.1% vs. 13.0%). In the multivariance analysis, subclinical hypothyroidism (aPR = 2.22 95% CI [1.47–3.36]) was independent factor associated with inadequate glycemic control (*p* < 0.001).

**Conclusions:**

A significant association was detected between subclinical hypothyroidism and inadequate glycemic control in older adults who presented at the “Centro Médico Naval” from 2010 to 2015.

## 1. Introduction

An older adult is more predisposed to acquire metabolic disorders due to multiple factors. For example, the elderly have greater prevalence of systemic chronic inflammatory reaction, oxidative stress, damaged DNA, cellular senescence, tissular dysfunction, and decreased mitochondrial dysfunction [1].

Additionally, aging is associated with deterioration of insulin secretion by beta cells in response to endogenous incretin hormones, which in turn is associated with a reduction in insulin sensitivity and promotes beta cell death by inducing mitochondrial dysfunction. Furthermore, there is a decrease in preadipocyte replication with expansion of senescent cells in the adipose tissue that increases lipotoxicity and favors a proinflammatory state [2]. Aging is also associated with changes in corporal composition and an increase in fatty mass, especially the visceral type, combined with a decrease of lean and skeletal mass [3], all of which causes a gradual intolerance to glucose and poor glycemic control.

Alterations in thyroid hormones have repercussions on the metabolism. For example, in hypothyroidism there is a decrease in insulin production by beta cells, and muscle cells develop less insulin sensitivity. Subclinical hypothyroidism shows an increase in insulin resistance due to a decrease in glucose transportation by the glucose 2 transporter (GLUT2) caused by a translocation of the GLUT 2 gene. Moreover, DM is a risk factor for thyroid disease because it alters thyroid-stimulating hormone (TSH) levels and the conversion of T4 to T3 in peripheral tissues. Hyperinsulinemia and insulin resistance can cause a proliferation of thyroid tissue, increasing the risk of developing lymphatic nodules and goiter [4].

In studies restricted to older persons, the reported prevalence of overt hypothyroidism has ranged between 0.2–5.7% and subclinical hypothyroidism between 1.5% and 12.5% of patients, making it the most prevalent endocrine disease among the aged, with a mayor prevalence in the female population [5]. In more recent studies in Europe, the prevalence of hypothyroidism was 4.7%, 4.11% for subclinical and 0.65% for overt hypothyroidism, with women over 65 years of age being the most affected [6]. This high prevalence can be explained by a decrease in organification and absorption of iodine and altered response to TSH in the elderly. In addition, changes in bioactivity, the sensitivity of thyrocytes to TSH hormone thyroid metabolism, and in the receptors and cofactors that modulate response to the influx of T3 cells have been described. In other words, these processes lead to a decrease in thyroid hormone production [7].

Regarding the variables related to thyroid function and glycemic control, it has been observed that among people with diabetes, females taking antihypertensive medications, who have a low educational level, are associated with thyroid dysfunction [8]. Additionally, DM type 2 patients that also have subclinical hypothyroidism have a greater risk of developing complications like cardiovascular disease [8, 9] and proliferative diabetic retinopathy [10].

Determining if there is an association between subclinical hypothyroidism and glycemic control in the elderly is relevant because it can lead to a better understanding of the pathogenesis and treatment of thyroid disease and diabetes. Complications may be prevented with better control.

The present study, therefore, aimed to determine if there is an association between subclinical hypothyroidism and glycemic control in older adults who presented at the Medical Navy Center of Peru between the years of 2010 and 2015.

## 2. Methods

### 2.1. Study Design

This study was a retrospective analytical study including a secondary analysis of the database of a study conducted in a Medical Navy Center (CEMENA) located in the Bellavista-Callao district in Peru, in which retired military personnel and their direct family members reside and receive care. The data were obtained from a cohort of adults older than 60 years of age from 2010 to 2015. The study was approved by the Ethics Committee of the Cientifica del Sur University (No. 113-CIEI-CIENTIFICA-2019, and registry code: 178-2019-PRE15). Participant anonymity was maintained and only necessary information was used to conduct the study.

### 2.2. Setting and Participants

The original population of the study was comprised of 1987 participants over the age of 60 years who were treated on an outpatient basis in the Geriatrics Service of the Naval Medical Center “Cirujano Mayor Santiago Távara” from 2010 to 2015. The participants were divided into 2 groups: first, older adults with inadequate glycemic control, and second, older adults with adequate glycemic control. The geriatric patients with a preceding diagnosis of primary or secondary hypothyroidism, DM, and the ones that did not have enough information for the variable of interest (glycemic control) were excluded. The final sample was comprised of 1385 older adults.

### 2.3. Data Collection Process

In the present study, the dependent variable was glycemic control and was defined as glycemic monitoring based on the evaluation of glycosylated hemoglobin (HbA1c) values and fasting glucose at baseline. Good glycemic control was considered with fasting glucose values between 90 and 130 mg/dL (5.0–7.2 mmol/L) and glycosylated hemoglobin levels ≤7.5%. Inadequate glycemic control was considered if any of these values were altered. The independent variable was subclinical hypothyroidism, which was defined as TSH concentrations above the upper normal limit together with free T4 concentrations within the reference range. Reference values were between 5 and 15 mU/L for TSH and between 0.7 and 1.8 ng/dL for free T4 [11, 12].

The analyzed variables were age; sex; education; marital status; body mass index (BMI), weight in kilograms divided by the square of height in meters; *comorbidities*, the coexistence of two or more diseases in the same person, for example, renal chronic disease with or without controls, arterial hypertension with treatment or recently diagnosed; *polypharmacy*, consumption of various drugs at the same time, i.e., more than 5 drugs were considered.

### 2.4. Statistical Analysis

The statistical analysis was performed using the statistical program STATA version 16. Descriptive statistics were used to determine percentages. The chi-square test was used to analyze potential associations between patient characteristics (age, sex, education, marital status, BMI, comorbidities, and polypharmacy) and glycemic control in the bivariate analysis. Finally, for the multivariate analysis, the Poisson regression with robust variance was used to determine if there is an association between the characteristics of older adults and glycemic control. The prevalence ratio (PR) was calculated with a confidence interval of 95% (95% CI).

## 3. Results

In this study, initially 1987 participants were selected; however, the final study sample comprised of data from 1385 older adults as those individuals that did not meet the inclusion criteria were excluded. Regarding the general characteristics, 45.6% of participants were between 71 and 80 years old; 58.4% were females; 65.3% were married; 43.8% had a normal BMI; 60.8% had arterial hypertension; 5.3% had chronic kidney disease; 29.5% had polypharmacy; and 15.2% had subclinical hypothyroidism (Table 1).

Among the elderly studied, 8.1% had inadequate glycemic control, and 91.9% had adequate glycemic control. Significant differences were found with respect to sex, marital status, BMI, arterial hypertension, chronic kidney disease, polypharmacy, and subclinical hypothyroidism between the two study groups (adequate versus inadequate glycemic control) (Table 2). In the first group, 72.3% were females, compared to only 57.4% in the second group. The proportion of married older adults was greater in the first group (83.3% vs. 71.2%, respectively), while the proportion of widowers was smaller in this group (10.2% vs. 21.5%). On the other hand, the proportion of the elderly with normal weight was greater in the first group (57.4% vs. 50.0%), while the proportion of older adults who were overweight was lower (13.9% vs. 28.9%). The proportion of older adults with chronic kidney disease (18.8% vs. 4.1%), polypharmacy (96.4% vs. 25.0%), and subclinical hypothyroidism (41.1% vs. 13.0%) was greater in the first group compared with the second group. Finally, the proportion of arterial hypertension was lower in the first group (52.7% vs. 62.1%, respectively).

In the multivariance analysis, female sex (aPR = 1.71 95% CI [1.11–2.62]), low weight (aPR = 1.67 95% CI [1.13–2.45]), polypharmacy (aPR = 5.98 95% CI [2.39–11.14]), subclinical hypothyroidism (aPR = 2.22 95% CI [1.47–3.36]), married (aPR = 0.59 95% CI [0.37–0.94]), widowed (aPR = 0.25 95% CI [0.13–0.50]), and overweight (aPR = 0.36 95% CI [0.21x0.61]) were independent factors associated with inadequate glycemic control (Table 3).

## 4. Discussion

The present study found an association between subclinical hypothyroidism and inadequate glycemic control. These results are similar to those reported in previous studies [13–15]. This relationship may be explained by the mechanisms of thyroid dysfunction such as alteration of the expression of a group of genes that cause physiological anomalies and decrease transport of glucose to cells, decrease absorption of splanchnic glucose, and increase release of glucose by the liver. Moreover, subclinical hypothyroidism can cause insulin resistance due to a translocation of the gene that encodes GLUT2 [4].

A considerable proportion of the aged was found to be overweight and/or obese, or had arterial hypertension, chronic renal disease, polypharmacy, and/or subclinical hypothyroidism. These findings may be explained by an increase in chronic comorbidities and polypharmacy present in older adults due to physiological, cultural, and socioeconomic changes [16]. Older adults have a greater risk of acquiring subclinical hypothyroidism due to a reduction in thyroid hormones caused by physiological changes, such as an alteration of the absorption and organification of iodine, a decrease in the sensitivity of thyrocytes to TSH, and pathological changes including comorbidities such as diabetes mellitus [2, 3, 7]. In the present study, the prevalence of subclinical hypothyroidism was 15.2%, which is greater than what has been reported in other studies [6, 17].

Additionally, 8.1% of the older adults in the present study had inadequate glycemic control. Physiological changes that occur in older adults may explain the inadequate glycemic control, including a greater composition of fatty mass with an increase in adipocyte inflammation, a reduction in lean mass, an increase in chronic systemic inflammation with oxidative stress, and a decrease in beta cells and their response to incretins which in turn induces an increase in insulin resistance [2, 3].

Participants who were overweight had a lower probability of having inadequate glycemic control, while those with low weight had a greater risk of presenting inadequate glycemic control. These results differ from those of other studies which found that an increase in BMI was associated with poor glycemic control due to hyperinsulinemia and insulin resistance which are related to obesity [18]. Furthermore, blood glucose levels may be more difficult to control in obese people [19]. There were other studies that had similar results to those of the present study, which showed that an increase in BMI is associated with a lower HbA1c. This finding may be attributed to the fact that weight loss caused by accumulated chronic metabolic inflammation and certain behaviors, such as smoking, can alter pancreatic beta cells and increase HbA1c [20].

Polypharmacy can be associated with poor adherence to treatment, with a decrease in hypoglycemic medication consumption and poor glycemic control [18]. Some studies have described a rate of therapeutic compliance less than 50% in patients with DM type 2 [21].

It is possible that polypharmacy could be a risk factor for hypothyroidism given that it was present in the majority of the participants (96.4%). The medications most associated with altered thyroid function include amiodarone, which can be administered for arrhythmias, a common condition in the elderly [22]. Other medications include lithium, antidepressants, antiepileptics, and rifampicin [23].

Similar to DM as the principal cause of inadequate glycemic control, there are other comorbidities that could alter glycemia such as high BMI, medications like corticosteroids, estrogens, and phenytoin, as well as conditions like acromegaly and Cushing's syndrome [24–26].

According to the present findings that subclinical hypothyroidism is associated with inadequate glycemic control in the elderly are important because subclinical hypothyroidism is difficult to diagnose due to its subtle clinical manifestations. Moreover, in elderly with multiple comorbidities the manifestations may be masked or go undetected. Nevertheless, even though hypothyroidism can have imperceptible clinical manifestations, it can have severe health consequences. Thus, these results suggest the need for screening for one of these pathological conditions in the aged when the other condition is present, to achieve an early diagnosis and apply interventions for their control to minimize consequences.

One of the main limitations of this study was the limited studies performed on a national level, and consequently, a lack of data with which to compare the results found in this investigation. Moreover, the population studied was derived from a small sector (Naval Medical Center) that may not reflect the prevalence of the country. Furthermore, it is not possible to determine any causal relationships with the findings in this study; so, implications should be taken cautiously. However, this study contributes findings that may have clinical relevance to the scientific community and may motivate future investigations from different institutional and population samples.

In conclusion, a significant statistical association was found between the presence of subclinical hypothyroidism and inadequate glycemic control. We recommend performing similar investigations in populations from other regions and institutions to obtain a better picture at the national level.

## Figures and Tables

**Table 1:** General characteristics of the study sample.

General data	*n* = 1,385	%
Age		
60–70 years	215	15.5
71–80 years	632	45.6
>80 years	538	38.8
Sex^1^		
Female	809	58.4
Male	571	41.2
Education^1^		
Education (≤11 years)	906	65.4
Education (>11 years)	384	27.7
Marital Status^1^		
Single	39	2.8
Married	904	65.3
Widowed	257	18.6
Divorced	52	3.8
Body mass index^1^		
Low weight	50	3.6
Normal weight	606	43.8
Overweight	330	23.8
Obese	210	15.2
Hypertension^1^		
Yes	842	60.8
No	530	38.3
Chronic kidney disease		
Yes	73	5.3
No	1,312	94.7
Polypharmacy^1^		
Yes	409	29.5
No	909	65.6
Subclinical hypothyroidism		
Yes	211	15.2
No	1,174	84.8

^1^The data did not add up to 1385 due to missing information.

**Table 2:** General characteristics of the sample according to glycemic control.

General data	Inadequate glycemic control *n* = 112 (8.1%)		Adequate glycemic control *n* = 1,273 (91.9%)		*p* ^2^
	*n*	%	*n*	%	
Age					
60–70 years	11	9.8	204	16.0	0.140
71–80 years	59	52.7	573	45.0	
>80 years	42	37.5	496	39.0	
Sex^1^					
Female	81	72.3	728	57.4	0.002
Male	31	27.7	540	42.6	
Education^1^					
Education (≤11 years)	76	70.4	830	70.2	0.974
Education (>11 years)	32	29.6	352	29.8	
Marital status^1^					
Single	5	4.6	34	3.0	0.014
Married	90	83.3	814	71.2	
Widowed	11	10.2	246	21.5	
Divorced	2	1.9	50	4.4	
Body mass index^1^					
Low weight	17	16.8	33	3.0	<0.001
Normal weight	58	57.4	548	50.0	
Overweight	14	13.9	316	28.9	
Obese	12	11.9	198	18.1	
Hypertension^1^					
Yes	59	52.7	783	62.1	0.049
No	53	47.3	477	37.9	
Chronic kidney disease					
Yes	21	18.8	52	4.1	<0.001
No	91	81.3	1,221	95.9	
Polypharmacy^1^					
Yes	108	96.4	301	25.0	<0.001
No	4	3.6	905	75.0	
Subclinical hypothyroidism					
Yes	46	41.1	165	13.0	<0.001
No	66	58.9	1,108	87.0	

^1^The data did not add up to 1385 due to missing information. ^2^Chi square test.

**Table 3:** Poisson regression.

Variables	Crude model PR (95% CI)	Adjusted model^1^ PR (95% CI)
Female sex	1.84 (1.24–2.75)	1.71 (1.11–2.62)
Marital status		
Single	Reference	Reference
Married	0.78 (0.33–1.80)	0.59 (0.37–0.94)
Widowed	0.33 (0.12–0.91)	0.25 (0.13–0.50)
Divorced	0.30 (0.66–1.47)	0.62 (0.23–1.66)
Body mass index		
Normal	Reference	Reference
Low weight	3.55 (2.25–5.61)	1.67 (1.13–2.45)
Overweight	0.44 (0.25–0.78)	0.36 (0.21–0.61)
Obese	0.60 (0.33–1.09)	0.93 (0.58–1.48)
Hypertension	0.70 (0.50–1.01)	1.31 (0.94–1.83)
Chronic renal disease	4.15 (2.75–6.26)	1.09 (0.61–1.60)
Polypharmacy	6.01 (2.22–7.35)	5.98 (2.39–11.14)
Subclinical hypothyroidism	3.88 (2.74–5.48)	2.22 (1.47–3.36)

PR: prevalence ratio, CI: confidence interval. ^1^Model adjusted for all covariables present in the table. Calculated by Poisson regression. Association between general characteristics and glycemic control in older adults from the naval medical center 2010–2015.

## Data Availability

The data used to support the findings of this study are included within the article.

## References

[1] Zhang K., Ma Y., Luo Y. (2023). Metabolic diseases and healthy aging: identifying environmental and behavioral risk factors and promoting public health. *Frontiers Public Health*.

[2] Chia C.W., Egan J.M., Ferrucci L. (2018). Age-related changes in glucose metabolism, hyperglycemia, and cardiovascular risk. *Circulation Research*.

[3] de Jaeger C. (2018). Physiology of aging EMC-kinesiterapia-med. *EMC-Kinesiterapia-Medicina Física*.

[4] Mohammed Hussein S.M., AbdElmageed R.M. (2021). The relationship between type 2 diabetes mellitus and related thyroid diseases. *Cureus*.

[5] Leng O., Razvi S. (2019). Hypothyroidism in the older population. *Thyroid Research*.

[6] Mendes D., Alves C., Silverio N., Batel Marques F. (2019). Prevalence of undiagnosed hypothyroidism in europe: a systematic review and meta-analysis. *European Thyroid Journal*.

[7] Calsolaro V., Niccolai F., Pasqualetti G. (2019). Overt and subclinical hypothyroidism in the elderly: when to treat?. *Frontiers Endocrinology*.

[8] Molla A., Habte F.B., Yimer R.M. (2022). Determinants of thyroid dysfunction among type 2 diabetes patients attending private hospitals in Dire Dawa, Eastern Ethiopia.

[9] Marushchak M., Vivsiana I., Musiienko V., Krynytska I., Kozak K. (2022). Subclinical hypothyroidism as a contributor to macrovascular complications in patients with type 2 diabetes mellitus. *Acta Clinical Croat*.

[10] El-Sehrawy A.A., Elkhamisy E.M., Badawi A.E. (2022). Subclinical hypothyroidism in patients with diabetic retinopathy: role ofvascular endothelial growth factor. *Endocrine, Metabolic & Immune Disorders-Drug Targets*.

[11] Malvetti Maffei M.V., Báez Cabral S.A., Santa Cruz FV (2016). Thyroid dysfunction in patients with type 2 diabetes mellitus. *Revista Virtual de la Sociedad Paraguaya de Medicina Interna*.

[12] García B. (2013). Endocrine diseases in elderly. *Revises Médica Clínica Las Condes*.

[13] Elgazar E H, Esheba NE, Shalaby S A, Mohamed WF (2019). Thyroid dysfunction prevalence and relation to glycemic control in patients with type 2 diabetes mellitus. *Diabetes & Metabolic Syndrome: Clinical Research & Reviews*.

[14] Chen R.H., Chen H.Y., Man K.M. (2019). Thyroid diseases increased the risk of type 2 diabetes mellitus: a nation-wide cohort study. *Medicine (Baltimore)*.

[15] Díez J, Iglesias P (2023). Prevalence of diabetes in people with thyroid dysfunction. *Medicina Clínica*.

[16] Soler P.A. (2020). *Medicina geriátrica: Geriatric medicine: A problem-based approach*.

[17] Hollowell J, Staehling N, Flanders W (2002). Serum TSH, T_4_, and thyroid antibodies in the United States population (1988 to 1994): national health and nutrition examination survey (NHANES III). *The Journal of Clinical Endocrinology & Metabolism*.

[18] Sisodia R.K., Chouhan M. (2019). The study of correlation between Body Mass Index and glycemic control-HbA1c in diabetes type 2 patients. *Internal Journal Advances Medicine*.

[19] Kennedy-Martin T., Boye K.S., Kennedy-Martin M. (2009). The association between body mass index and glycemic control in patients with type 2 diabetes across eight countries: a literature review. *Current Research Diabetes Obes Journal*.

[20] Zhang Y., Guo Y., Shen X., Zhao F., Yan S. (2019). Lower body mass index is not of more benefit for diabetic complications. *Journal of Diabetes Investigation*.

[21] Rojas-Gómez R., Rojas-Gómez E. (2018). Polifarmacia y adherencia terapéutica en el adulto mayor con Diabetes Mellitus. *Internal Journal Medical*.

[22] Yokoyama S., Tanaka Y., Hosomi K., Takada M. (2021). Polypharmacy Is Associated With Amiodarone-Induced Hypothyroidism. *Internal Journal Medical Science*.

[23] Montanelli L., Benvenga S., Hegedüs L., Vitti P., Latrofa F., Duntas L. H., Vitti P., Hegedüs L. (2018). Drugs and Other Substances Interfering with Thyroid Function. *Thyroid Diseases Pathoglogy Diagnosis Treatment*.

[24] Mouri Mi., Badireddy M. (2023). *Hyperglycemia, in: StatPearls*.

[25] Liu X., Zhu X., Miao Q., Ye H., Zhang Z., Li Y.-M. (2014). Hyperglycemia induced by glucocorticoids in nondiabetic patients: a meta-analysis. *Annals Nutrition Metabolism*.

[26] Oka R., Yagi K., Hayashi K. (2014). The evolution of non-diabetic hyperglycemia: a longitudinal study. *Endocronlogy Journal*.

[27] Quintanilla K., Joo K.M., La Torre H.L. (2023). Association between subclinical hypothyroidism and glycemic control in older adults in a medical center in Peru.

